# Erratum: A TetR-family protein (CAETHG_0459) activates transcription from a new promoter motif associated with essential genes for autotrophic growth in acetogens

**DOI:** 10.3389/fmicb.2024.1415945

**Published:** 2024-04-22

**Authors:** Frontiers Production Office

**Affiliations:** Frontiers Media SA, Lausanne, Switzerland

**Keywords:** *Clostridium autoethanogenum*, Wood–Ljungdahl pathway, transcriptional regulation, gas fermentation, autotrophy

Due to a production error, an incorrect accession number, PXD0014421, was included in the published article's Data Availability Statement. The correct accession number is PXD014421.

The publisher apologizes for this mistake. The original article has been updated.

