# Supplementary figures and images for: A TetR-Family Protein (CAETHG_0459) Activates Transcription From a New Promoter Motif Associated With Essential Genes for Autotrophic Growth in Acetogens
Source: Front Microbiol. 2019 Nov 15;10:2549. doi: 10.3389/fmicb.2019.02549 (PMC6873888; doi:10.3389/fmicb.2019.02549)

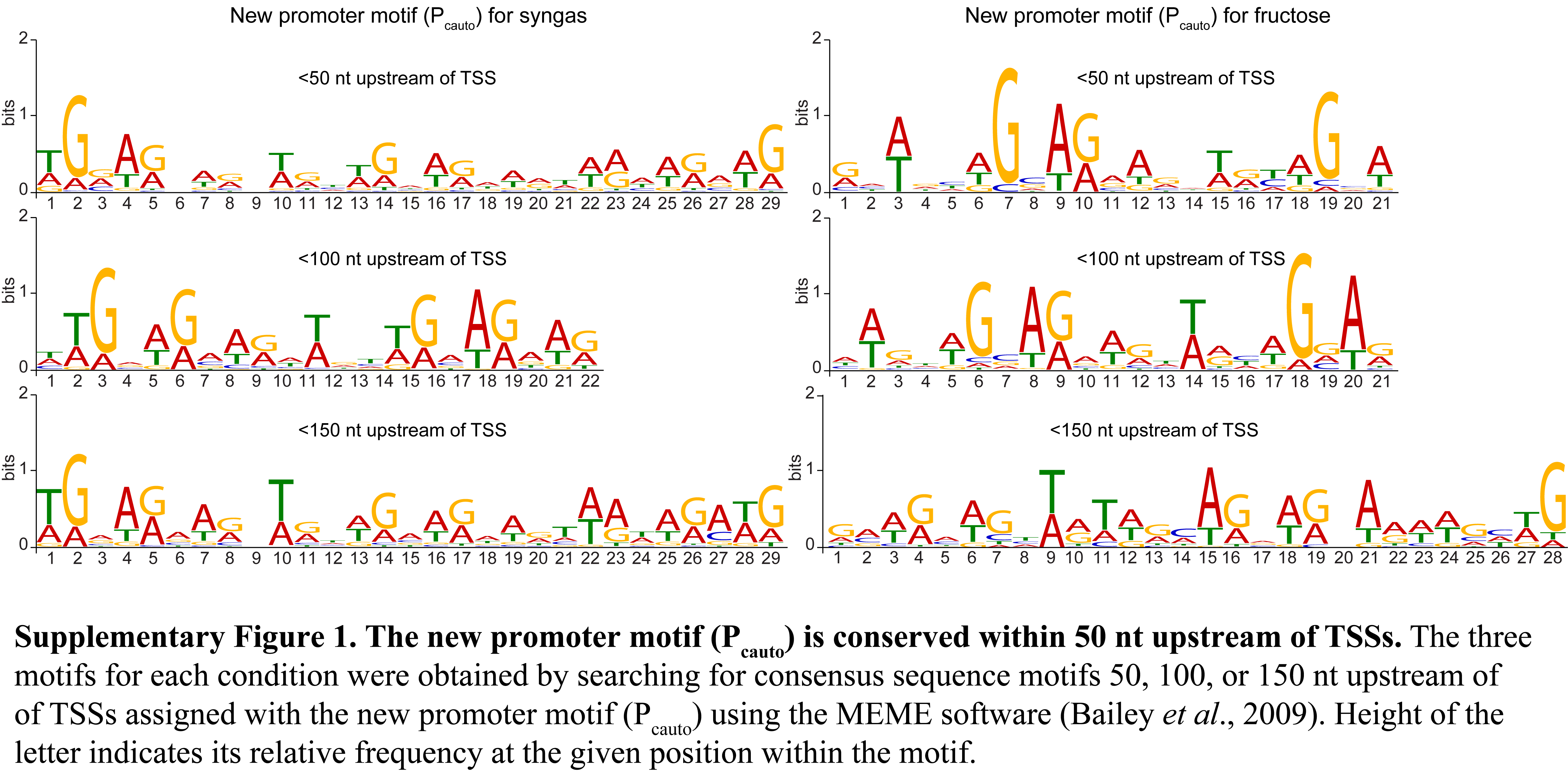

Supplement: Supplementary file 1 [file Image_1.TIF]
