# Supplementary material for: A TetR-Family Protein (CAETHG_0459) Activates Transcription From a New Promoter Motif Associated With Essential Genes for Autotrophic Growth in Acetogens
Source: Front Microbiol. 2019 Nov 15;10:2549. doi: 10.3389/fmicb.2019.02549 (PMC6873888; doi:10.3389/fmicb.2019.02549)
Supplement: Supplementary file 2 [file Image_2.pdf]

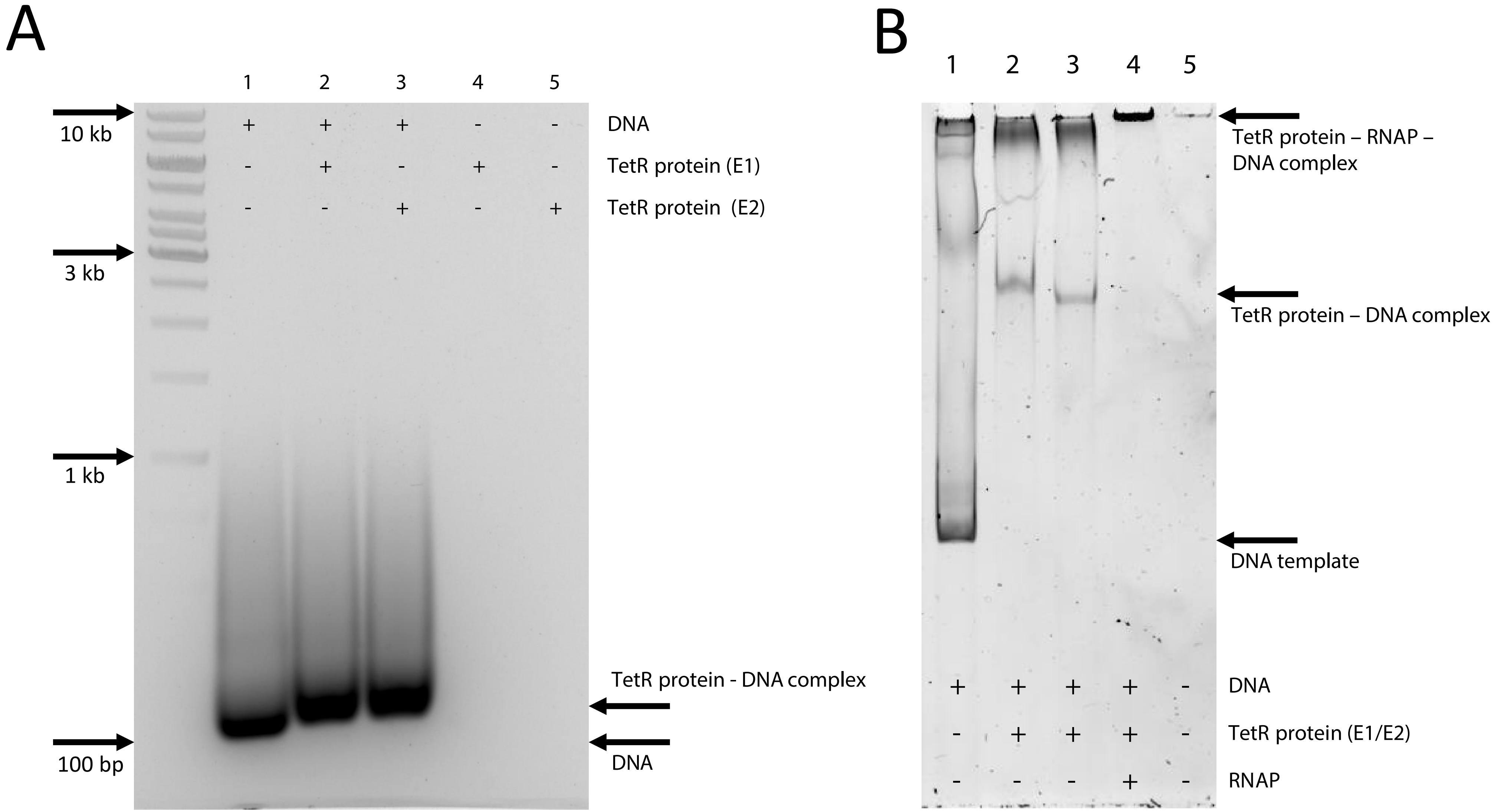

Supplementary Figure 2. EMSA experiments of DNA-protein interaction of the TetR-family protein (CAETHG_0459) and the new promoter motif (P_{cauto}) using gel electrophoresis. (A) EMSA in 1 % agarose gel. Two different TetR-family protein (CAETHG_0459) extracts were tested (E1, E2). The first lane corresponds to a 1 kb gene ruler. **(B)** EMSA in 7.5 % polyacrylamide gel. The combination of proteins and DNA is shown for each lane: present (+), absent (-). See Methods for experimental details.
