# Supplementary material for: A TetR-Family Protein (CAETHG_0459) Activates Transcription From a New Promoter Motif Associated With Essential Genes for Autotrophic Growth in Acetogens
Source: Front Microbiol. 2019 Nov 15;10:2549. doi: 10.3389/fmicb.2019.02549 (PMC6873888; doi:10.3389/fmicb.2019.02549)
Supplement: Supplementary file 4 [file Table_2.DOC]

| **Supplementary File 2.** Molecular cloning. Bacterial strain, plasmids and primers used* | | |
| --- | --- | --- |
|  |  |  |
| **Bacterial strain** | **Description** | **Reference** |
|  |  |  |
| *Escherichia coli* BL21 | Expression host | Bioline |
| *Escherichia coli* DH5α | Cloning host | Bioline |
|  |  |  |
| **Plasmid** | **Description** | **Reference** |
|  |  |  |
| pBR322 | Cloning vector | Life Technologies |
| pACYC184 | Expression vector | Lab collection |
| pET28a+ | Expression vector | Lab collection |
| pBR_PprpR-GFPUV | pBR322 derived plasmid. GFP-uv expression plasmid under the control of prpR promoter | Lab collection |
| pET_SelT | pET28a+ derived plasmid. KanR, *T7-SelT* | This work |
| pET_TetR1^a^ | pET28a+ derived plasmid. KanR, *T7-TetR1* | This work |
| pET_TetR2 ^b^ | pET28a+ derived plasmid. KanR, *T7-TetR2* | This work |
| pET_GntR | pET28a+ derived plasmid. KanR, *T7-GntR* | This work |
| pET_Sigma70 | pET28a+ derived plasmid. KanR, *T7-Sigma70* | This work |
| pBR_PWLP_GFP | pBR322 derived plasmid. AmpR, *P_WLP_-gfpUV* | This work |
| pAC_PWLP_GFP | pACYC184 derived plasmid. CmR, *P_WLP_-gfpUV* | This work |
|  |  |  |
| **Primer name** | **Sequence** | **Target region** |
|  |  |  |
| SelT_FWD | ctggtgccgcgcggcagccatATGGATAAAAAACAATTATTAAGAAAAC | SelT (CAETHG_2839) |
| SelT_REV | ggtgctcgagtgcggccgcaagcttattaCTAAAACTCTGTAAAAGCATCTACC |
| TetR2_FWD ^b^ | ctggtgccgcgcggcagccatATGGCTCAAATAAAAAAAGAC | TetR2 (CAETHG_0936) |
| TetR2_REV ^b^ | ggtgctcgagtgcggccgcaagcttattaTTACTTAGTGTATTCTGTAAGTAATCTTTTAAATC |
| GntR_FWD | ctggtgccgcgcggcagccatATGTCATCGCAAAATGTTAC | GntR (CAETHG_3915) |
| GntR_REV | ggtgctcgagtgcggccgcaagcttattaCTAATTATTATCTCCATAAAGAAATTTTTTG |
| CautoPnew_FWD | ttgacagcttatcatcgataagcttAATTAGGCGTAAATGTAAGATTATC | P_WLP_ |
| CautoPnew_REV | ctttactcatATTGTTTCCTCCTAAATGTTTTTAG |
| Cauto_gfp_FWD | aggaaacaatATGAGTAAAGGAGAAGAACTTTTC | gfp-UV |
| Cauto_gfp_REV | gtgataaactaccgcattaaagcttattaTTATTTGTAGAGCTCATCCATG |
| pET_conf(FWD) | GATATAGGCGCCAGCAACC |  |
| pET_conf(REV) | AGCCAACTCAGCTTCCTTTC |  |
| PWLP_GFP_conf(FWD-1) | TGTCTCATGAGCGGATACATATT |  |
| PWLP_GFP_conf(REV-1) | GTCTTGTAGTTCCCGTCATCTT |  |
| PWLP_GFP_conf(FWD-2) | GGAAACATTCTCGGACACAAAC |  |
| PWLP_GFP_conf(REV-2) | ATACCCACGCCGAAACAA |  |
| PWLP_GFP_conf(FWD-3) | GGAAACATTCTCGGACACAAAC |  |
| PWLP_GFP_conf(REV-3) | TTGTTTCGGCGTGGGTAT |  |
| * Upper cases indicate the region homologous to the target region during the PCR amplification. Lower cases indicate homologous region for Gibson assembly.  ^a^ TetR 1: CAETHG_0459  ^b^ TetR 2: CAETHG_0936 | | |
